# Critical *in vivo* roles of WNT10A in wound healing by regulating collagen expression/synthesis in *WNT10A*-deficient mice

**DOI:** 10.1371/journal.pone.0195156

**Published:** 2018-03-29

**Authors:** Ke-Yong Wang, Sohsuke Yamada, Hiroto Izumi, Manabu Tsukamoto, Tamiji Nakashima, Takashi Tasaki, Xin Guo, Hidetaka Uramoto, Yasuyuki Sasaguri, Kimitoshi Kohno

**Affiliations:** 1 Shared-Use Research Center, School of Medicine, University of Occupational and Environmental Health, Kitakyushu, Japan; 2 Department of Pathology and Laboratory Medicine Kanazawa Medical University, Ishikawa, Japan; 3 Department of Occupational Pneumology, School of Medicine, University of Occupational and Environmental Health, Kitakyushu, Japan; 4 Department of Orthopaedic Surgery, School of Medicine, University of Occupational and Environmental Health, Kitakyushu, Japan; 5 Department of Human, Information and Life Sciences School of Medicine, University of Occupational and Environmental Health, Kitakyushu, Japan; 6 Department of Pathology and Cell Biology, School of Medicine, University of Occupational and Environmental Health, Kitakyushu, Japan; 7 Department of Thoracic Surgery, Kanazawa Medical University, Ishikawa, Japan; 8 Laboratory of Pathology, Fukuoka Tokushukai Hospital, Fukuoka, Japan; 9 Asahi-Matsumoto Hospital, Kitakyushu, Japan; Northwestern University, UNITED STATES

## Abstract

**Background:**

We have reported that WNT10A plays a critical role in the growth of fibroblasts/myofibroblasts and microvascular endothelial cells, i.e.; wound healing/scarring. To ascertain the *in vivo* regulatory, central functions of WNT10A, we examined the net effects of WNT10A depletion using *WNT10A*-deficient mice (*WNT10A*^–/–^).

**Methods and results:**

We generated *WNT10A*^–/–^mice, displaying a range of unique phenotypes of morpho/organogenetic failure, such as growth retardation, alopecia, kyphosis and infertility, and then focused on the functions of WNT10A in wound healing. We subjected C57BL/6J wild-type (WT) or *WNT10A*^–/–^mice to skin ulcer formation. The *WNT10A*^–/–^mice had significantly larger injured areas and delayed wound healing, which were associated with (a) a smaller number of fibroblasts/myofibroblasts and microvessels; and (b) more reduced expression and synthesis of collagen, compared with WT mice with intact WNT10A expression, especially in those with activated myofibroblasts.

**Conclusions:**

These observations indicate that WNT10A signaling can play a pivotal in vivo role in wound healing by regulating the expression and synthesis of collagen, as one of fibrogenic factors, at least in part, and critical *in vivo* roles of WNT10A-mediated effective wound healing are extremely closely associated with collagen expression.

## Introduction

It has been suggested that mechanical stress elicits tissue injury/wounds and subsequent tissue repair and fibrosis, i.e., wound healing/scarring, along with the production of extracellular matrix (ECM) including collagen, through mechanisms driven by WNT signaling [1, 2]. Furthermore, since wound healing is an essential trans-differentiation process of epithelial-mesenchymal transition, which plays a pivotal role in initiating the progression of malignant neoplasms, at least in part, WNT signaling has a close correlation with the tumoral stroma microenvironment and cancer growth [3]. Indeed, Dvorak once famously said, “Tumors are ‘wounds’ that do not heal [4].”

There are cellular parallels between wound granulation and tumor stroma in the phase of tissue remodeling, which consists of a large number of migrating/proliferating fibroblasts and myofibroblasts and newly formed blood vessels admixed with inflammatory cells, especially macrophages [5]. The main difference between ‘healing’ wounds and ‘unhealing’ tumors is that the processes of ECM production with collagen expression in malignant neoplasms are not self-limiting, activating the latent wound-healing program of the cancer-carrying host uniquely in an exaggerated and prolonged manner [5]. This is referenced in an old hypothesis of cancer as “an overhealing wound” [6].

WNT signaling is separated into two major pathways: ‘canonical’ (involving β-catenin) and ‘non-canonical’ (independent of β-catenin) ones [7, 8]. WNT members are ubiquitously expressed in humans, but the WNT signal transduction system is a highly complicated cascade critical to various pathophysiological actions [9, 10]. Among the *WNT* gene family members, *WNT10A* may play a central, key role in many processes of morpho/organogenesis or tumorigenesis in regulating fibrogenic factors, such as collagen [11–14]. Indeed, previous reports from our laboratories have shown that WNT10A overexpression induces the carcinomatous growth/proliferation of both microvascular endothelial cells and fibroblasts and/or myofibroblasts in *in vivo* nude mouse xenograft models [15]. Furthermore, WNT10A expression has been identified in human keloid dermal myofibroblasts, which immunohistochemically display the specific expression of α-smooth muscle actin (α-SMA), but not in normal skin dermal fibrocytes [15]. α-SMA-positive activated fibroblasts and/or myofibroblasts are known to be intimately involved in wound healing by secreting ECM, including several types of collagen [16]. We have more recently reported that WNT10A overexpression is closely associated with human clinical diseases of not only acute interstitial nephritis due to fibrosis but also acute exacerbation of idiopathic pulmonary fibrosis [17, 18].

Given these previous findings, we can readily hypothesize that WNT10A signaling plays a pivotal *in vivo* role in wound healing as well as tumor (i.e., ‘unhealing wound’) growth by stimulating ECM production. However, very few studies have investigated the direct relationships between the WNT10A signaling pathway and wound healing, despite the ubiquitous expression of WNT10A.

In the present study, we aimed to determine the *in vivo* net effects of WNT10A depletion and key factors of WNT10A in murine models of wound healing, with a particular focus on its functions in fibrogenesis with collagen expression/synthesis. We examined for the first time the crucial roles of WNT10A in ‘wound’ healing using mice genetically deficient in *WNT10A* (*WNT10A*^–/–^).

## Materials and methods

### Animals and generation of WNT10A-knockout (WNT10A^–/–^) mice

WNT10A targeted embryonic stem cells (ESCs) were obtained from the School of Veterinary Medicine, University of California, Davis (UC Davis, CA, USA); The knockout deleted 12663 bp (74791516 to 74804179 of mouse chromosome 1, genome build 38, Clone ID:14810A), which included the entire WNT10A coding region. The knockout construct replaced the *WNT10A* gene in ES cells (C57BL/6 background) with a ZEN-Ub1 cassette that introduced a bacterial lacZ code at the natural *WNT10A* translation initiation codon (in exon 1) and a downstream neomycin phosphotransferase gene (NEOr) driven by the human ubiquitin C gene promoter (hUBCpro). The neomycin resistance gene was bracketed by a locus of X-over P1 sequence from bacteriophage P1 (loxP) sequences for convenient removal of the NEOr selection code (S1 Fig). To generate chimeric mice, these ESC clones were microinjected into C57BL/6 blastocysts at NPO Biotechnology Research and Development, Osaka University (Osaka, Japan). Successful knockout was determined by reverse transcription polymerase chain reaction (RT-PCR) genotyping (http://www.velocigene.com/komp/detail/14810). Heterozygous *WNT10A*^+/–^females were then bred with *WNT10A*^+/–^males, and *WNT10A*^–/–^mice were born at the expected Mendelian ratio.

Experiments were performed in 10- to 12-week-old male *WNT10A*^–/–^mice, and wild-type (WT) C57BL/6J mice (Charles River, Yokohama, Japan) were used to provide a control group in some experiments (n = 8 to 12 mice per experiment). Animals were provided their diet and water *ad libitum* and maintained on a 12-h light/dark cycle. All animal experiments were conducted according to the Laboratory Animal Research Center at University of Occupational and Environmental Health School of Medicine. All surgery was performed under anesthetization using mixture of ketamine 50 mg/kg (Daiichi Sankyo Co., Tokyo, Japan) and medetomidine 1 mg/kg (Meiji Yakuhin Co., Tokyo, Japan) as previously described [15, 19–21].

The Ethics Committee of Animal Care and Experimentation, University of Occupational and Environmental Health (Japan) approved the protocols. They were performed according to the Institutional Guidelines for Animal Experiments and the Law (no. 105) and Notification (no. 6) of the Japanese Government. The investigation conformed to the Guide for the Care and Use of Laboratory Animals published by the US National Institutes of Health (NIH Publication No. 85–23, revised 1996).

### Body weight, bone size and bone mineral density (BMD)

The body weight of the mice was measured once a week, and the weights from 1 to 12 months were evaluated to determine the change in body weight. The right femur of 12-week-old mice was prepared by removing all of the soft tissue and then measured with digital calipers (Digimatic Caliper; Top Man, Hyogo, Japan). The length of the femur represented the distance between the top of the greater trochanter to the distal end of the lateral femoral condyle. We also measured the BMD (mg/cm^2^) in the right femur using dualenergy X-ray absorption (DXA; DCS-600; Aloka, Tokyo, Japan) [22].

### Micro-computed tomography (CT)

Mice were anesthetized with 0.3 mg/kg of medetomidine (Kyoritsu Seiyaku Corporation, Tokyo, Japan), 1.0 mg/kg of midazolam (Astellas Pharma Inc., Tokyo, Japan) and 5.0 mg/kg of butorphanol (Meiji Seika Pharma Co., Ltd., Tokyo, Japan) and subjected to whole-body three-dimensional (3D) micro-CT (CosmoScan GX; Rigaku, Tokyo, Japan) to identify the skeleton. In addition, CT images of the visceral and subcutaneous fat were acquired by micro-CT with a resolution of 120×120×120 μm^3^, a tube voltage of 90 kV and a tube current of 88 μA. The exposure time was 2 minutes. The CT images of body fat were visualized using the Analyze 12.0 software program (AnalyzeDirect, Inc., Overland Park, KS, USA) from the base of the ensiform cartilage to the pelvic floor [23].

### Murine model of wound healing

Ten-week-old male mice were anesthetized with a mixture of ketamine 50 mg/kg (Daiichi Sankyo Co.) and medetomidine 1 mg/kg, (Meiji Yakuhin Co.) and the dorsal skin was shaved to remove the hair. Two superficial wounds per mouse were created at dorsal sites using a 6-mm biopsy punch. Full-thickness skin was removed, exposing the underlying muscle. The wound area size was measured daily using the two principal perpendicular diameters. The average diameter was used to evaluate wound healing [24, 25]. At days 3, 7 and 10 post-injury (n = 8 per each group), the mice were killed in a fed state by intraperitoneal anesthetization with an overdose of ketamine (100 mg/kg) (Daiichi Sankyo Co.) and medetomidine (2 mg/kg) (Meiji Yakuhin Co.). The healing skin was formalin-fixed and embedded in paraffin for a histologic examination. Frozen sections of the healing skin were also produced for an immunofluorescence evaluation.

### Primary cells and culture conditions

Dermal fibroblasts were isolated from WT and *WNT10A*^–/–^mice at four weeks of age. To culture fibroblasts, a 2 × 2-cm piece of full thickness skin was taken from the euthanized mouse after removing the hair. The skin was then washed several times in PBS containing 1% penicillin and 1% streptomycin, and the hypodermis fat tissue was removed with a sterile blade under sterile conditions. The skin was cut into small pieces, and each piece was placed on a 25-mm culture dish with the dermis facing down. Small drops of FBS were placed on each piece of skin, and the dish was kept in a 37 °C incubator supplemented with 5% CO_2_ for 4 h. DMEM containing 10% FBS, 1% penicillin and 1% streptomycin was then added very slowly to the dish, and care was taken to avoid detachment of the pieces from the dish surface. After 1 to 2 weeks, the fibroblasts had migrated out of the skin and adhered to the dish surface [25]. The fibroblasts were also cultured on a chamber slide (ThermoFisher Scientific, Yokohama, Japan) for immunofluorescence staining.

Dermal fibroblasts isolated from WT or *WNT10A*^–/–^mice were cultured in growth medium (DMEM containing 10% FBS) until they formed confluent monolayers. In some cases, WT dermal fibroblasts were placed in growth medium containing anti-WNT10A antibody. Similarly, *WNT10A*^–/–^dermal fibroblasts were cultured in growth medium conditioned by WT dermal fibroblasts or growth medium containing anti-WNT10A antibody (dilution, 1:1000).

### Antibodies

The amino acid peptide corresponding to the WNT10A amino acid sequence at positions 160 to 172 was chemically synthesized and purified, and its portion was coupled to keyhole hemocyanin (KLH). Rabbits (Japanese White) were immunized with the peptide conjugated to KLH. On day 0, an initial immunization was administered with 400 μg of peptide conjugated to KLH in 1 mL of a 1:1 emulsification of the conjugated peptide and complete Freund's adjuvant at 4 separate sites. Subsequent immunization was given at 4 separate sites with 200 μg of peptide conjugated to KLH in 1 mL of a 1:1 emulsification with incomplete Freund's adjuvant at 2-week intervals (3 times in total). Antisera were obtained by bleeding the animals on day 56. The resulting antibody titers against the WNT10A peptide were determined by an enzyme-linked immunosorbent assay (ELISA). This antibody was used for immunohistochemical and immunofluorescence staining. To determine the number of myofibroblasts and microvessels in wound healing lesions and tumor tissue, immunohistochemical staining was performed with monoclonal mouse anti-human α-SMA (dilution 1:150; Dako, Tokyo, Japan) for myofibroblasts and mouse monoclonal anti-CD31 antibody (dilution 1:500; Dako) for endothelial cells. To further assess the importance of WNT signaling in wound healing regulation, β-catenin and WNT10A immunohistochemical staining were performed using rabbit polyclonal anti-β-catenin antibody (dilution 1:500, AC 121 °C; Abcam, Tokyo, Japan) and rabbit polyclonal anti-WNT10a antibody (dilution 1:5000). We further performed IHC using the rabbit polyclonal anti-Type I collagen (dilution 1:500; Abcam) and rabbit polyclonal anti-Type III collagen (dilution 1:200; Proteintech, Rosemont, IL, USA). Immunofluorescence staining of Type I collagen (dilution 1:500; Abcam) and Type III collagen (dilution 1:200; Proteintech) were then carried out, as previously described [26–28]. For double-immunofluorescence, the frozen sections of wound healing skin tissue at day 3 were labeled with mouse monoclonal α-SMA (dilution: 1:150; Dako) and rabbit polyclonal anti-WNT10A antibody (dilution 1:5000) and then visualized with goat anti-mouse IgG antibodies conjugated with Alexa Fluor Dyes (*red-stained*) and goat anti-rabbit IgG antibodies conjugated with Alexa Fluor Dyes (*green-stained*) (Invitrogen, Carlsbad, CA, USA); The frozen sections of wound healing skin tissue at day 3 were labeled with rabbit monoclonal anti-Histone H3 (phosphor S10) antibody (dilution 1:1000; Abcam, Tokyo, Japan) and then visualized with goat anti-rabbit IgG antibodies conjugated with Alexa Fluor Dyes (*green-stained*) (Invitrogen, Carlsbad, CA, USA), and incubated with DAPI (4’,6-Diamidino-2-Phenylindole, Dihydrochloride) (dilution: 1:10000; ThermoFisher) for nuclear staining. We used the HistoMouse Plus Kit (Invitrogen) to block endogenous mouse IgG [26, 27]. Stained tissues were placed on a glass slide, intimal side up, coverslipped by surface tension, and then viewed by confocal laser scanning microscopy (LSM5 Pascal Exciter; Carl Zeiss, Oberkochen, Germany) with ×40 UPlanApo oil immersion objectives. Mouse monoclonal antibodies to WNT10A and anti-β-actin (dilution, 1:5,000; Santa Cruz Biotechnology, Dallas, TX, USA) were used for Western blotting.

### Histopathology, immunohistochemistry (IHC) and immunofluorescence study

The mice of WT and *WNT10A*^–/–^were killed in a fed state by intraperitoneal anesthetization with an overdose of ketamine (100 mg/kg) (Daiichi Sankyo Co.) and medetomidine (2 mg/kg) (Meiji Yakuhin Co.), and all of organs were removed, formalin-fixed in 10% neutral buffered formalin for 24h, and embedded in paraffin for a histological examination. Tissue specimens were stained with hematoxylin and eosin (H&E) or Masson’s trichrome or prepared as immunohistochemistry (IHC) samples in sequential sections after fixation [15, 17–21, 26–27,29]. Frozen sections of healing skin were also produced for an immunofluorescence evaluation. The necrotic area/tumor area ratio was evaluated using an Olympus VS120 Virtual system (Olympus, Tokyo, Japan). The number of activated myofibroblasts and microvessels was counted in 10 randomly selected fields of sections of healing skin and tumor lesions, respectively (original magnification: x400), as previously described [26, 27]. The frozen sections of healing skin tissue and primary cells of dermal fibroblasts were rinsed with phosphate-buffered saline (PBS) and immediately fixed in 4% paraformaldehyde in 0.1 M PBS at pH 7.4 at room temperature.

All histological and IHC slides were evaluated by two independent observers (certified pathologists: Sohsuke Yamada and Ke-Yong Wang) who were blinded to the physical outcome or other biological and pathological data for each sample. In case of disagreement, a consensus score was determined by a third board-certified pathologist (Yasuyuki Sasaguri). Agreement between observers was excellent (> 0.9) for all sections investigated as measured by interclass correlation coefficients.

### Reverse transcriptase-polymerase chain reaction (RT-PCR) and Real-time RT-PCR

Total RNA was extracted with Trizol reagents (Invitrogen) from the skin of mice or Dermal fibroblasts isolated from the skin of mice sacrificed for the day-3 wound healing model after careful removal of blood cells, using the sham-operated skin as controls. All procedures were performed as described previously [19, 22, 24–25, 28, 30–31]. RNase-free conditions were used to prevent mRNA degradation. First-strand cDNA was synthesized with Superscript II RT (Invitrogen) using random primers according to the manufacturer’s instructions. The expression of the WNT10A gene was analyzed by RT-PCR and the relative amounts of PCR products were normalized by those of mGAPDH. Quantitative real-time RT-PCR was performed using the TaqMan fluorogenic probe method with a Step One real time PCR system (ThermoFisher Scientific). The specific primers and probes of collagen, Type I α1 (Mm00801666-g1; Applied Biosystems, Foster City, CA, USA) and Type III α1 (Mm01254476-m1; Applied Biosystems) were used.

Cycling conditions were as follows: 50 °C for 2 min, 95 °C for 10 min followed by 45 cycles of 95 °C for 15 s and 60 °C for 1 min. Threshold cycle (*C*_*T*_) values were measured corresponding to the cycle number at which the fluorescent emission, monitored in real time, reached a threshold of 10 standard deviations (SDs) above the mean baseline from cycles 1–15. Serial 1:10 dilutions of plasmid DNA containing each target cDNA were analyzed and served as standard curves from which the rate of change in the *C*_*T*_ was determined.

Copy numbers of target cDNA were estimated by standard curves. All reactions for samples were performed in triplicate. Mean data were obtained from the values of each reaction. To determine the mRNA levels of various genes, an mRNA expression index was used wherein the mRNA expression was standardized by 18s ribosomal RNA (rRNA) (Applied Biosystems). The mRNA expression index in arbitrary units (AU) was calculated as follows: mRNA expression index = (copy numbers of target gene mRNA / copy numbers of 18s rRNA) × 1 AU.

### Microarray studies and data analyses

DNA microarray analyses were performed using a 3D-Gene (Toray Industries, Kamakura, Kanagawa, Japan), as previously described [15, 27]. High-quality RNA samples (1 μg each) prepared from injured skin of WT and *WNT10A*^–/–^wound healing model mice sacrificed at day 3 were amplified and labeled with cyanine 3 (Cy3) and cyanine 5 (Cy5)-CTP (Amersham Biosciences Corp., Piscataway, NJ, USA) to produce labeled cRNA using Agilent low-RNA-input fluorescent linear amplification kits in accordance with the manufacturer's protocol. After amplification and labeling, the dye-incorporation ratio was determined using a Nanodrop spectrophotometer, and ratios were within 10 to 20 pM per μg of cRNA. For hybridization, 750 ng of Cy3-labeled cRNA and 750 ng of Cy5-labeled cRNA were fragmented and hybridized to an Agilent Technologies Mouse Gene Expression Microarray using the Agilent Gene Expression hybridization kit, as described in the Two-color Microarray-based Gene Expression Analysis Version 4.0 manual. After hybridization, the microarrays were washed and scanned with the Agilent dual-laser DNA microarray scanner. Scans were converted to data files using the Agilent Feature Extraction software program (Version 8.5). Data were deposited in a MIAME-compliant database (GSE23969) and analyzed with the Microsoft Access and Spotfire software programs. Arrays were scanned using an Agilent dual-laser DNA microarray scanner with SureScan technology, extracted by the feature Extraction software program, and analyzed by the Rosetta Resolver^®^ software program (Rosetta Biosoftware, Kirkland, WA, USA). Three replicate samples were used for each experiment. Genes were selected as significant based on the criterion of >2-fold (up- or down-regulated) change.

### Western blot analyses

Proteins (40 μg) isolated from the skin tissues of WT and *WNT10A*^–/–^mice were separated by sodium dodecyl sulfate-polyacrylamide gel electrophoresis (SDS-PAGE) and transferred to Immun-Blot polyvinylidene difluoride (PVDF) membranes (Bio-Rad Laboratories, K.K., Tokyo, Japan) using a semi-dry blotter. The blotted membranes were treated with 5% (w/v) skim milk in 10 mM Tris, 150 mM NaCl and 0.2% (v/v) Tween-20 and incubated for 1 h at room temperature with the primary antibody. The membranes were then incubated for 45 min at room temperature with a peroxidase-conjugated secondary antibody and visualized using an ECL kit (GE Healthcare Bio-Science, Buckinghamshire, UK). Bands on Western blots were analyzed densitometrically using the Scion Image software program (version 4.0.2; Scion Corp., Frederick, MD, USA).

### Statistical analyses

Results are expressed as the means ± standard error (SE). Significant differences were analyzed using Student’s *t*-test, Welch’s *t*-test or a one-way analysis of variance (ANOVA) where appropriate. In all cases in which an ANOVA was applied for non-parametric data, Tukey’s multiple comparison *post hoc* test was used [15, 26–29, 32]. Values of *P* < 0.05 were considered statistically significant.

## Results

### *WNT10A*^*–/–*^mice showed a range of unique phenotypes of morpho/organogenetic failure in a background of stromagenic failure

We generated *WNT10A*^–/–^mice with no expression of *WNT10A* in tissues, regardless of treatment, according to RT-PCR and western blotting (S2 and S3 Figs), whereas WT mice had normal expression of *WNT10A* mRNA.

**Fig 1:**
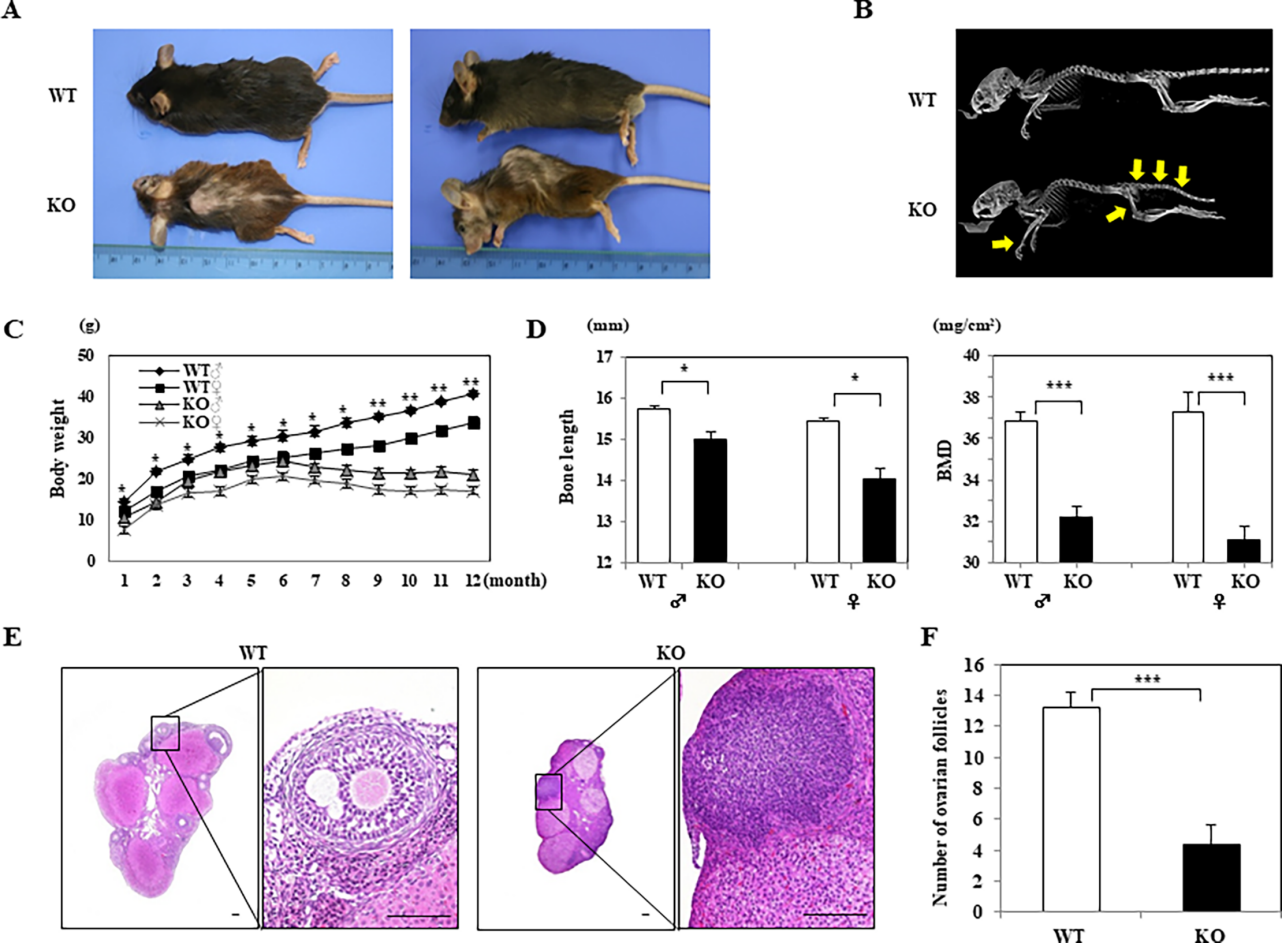
Our generated *WNT10A*^–/–^mice (KO) showing a range of unique phenotypes of morpho/organogenetic failure. **A)** Under basal conditions, morphological examinations between the 12-week-old wild-type (WT) and KO mice (n = 10 mice per group) show that the KO mice are grossly smaller in size and have marked alopecia and kyphosis compared to WT. **B)** Representative whole-body X-ray confirms significant growth retardation in the KO mice, accompanied by marked kyphosis and many bony hyperlucent areas (arrows), in contrast to WT mice (n = 10 mice per group). **C)** From 1 to 12 months of age (whole one year), both male and female KO display significantly smaller body weights, as reflected by more essential growth retardation, than WT (n = 10 mice per group). **D)** The bone length and bone mineral density (BMD) in both male and female 12-week-old KO mice are significantly lower than those of WT mice (n = 10 mice per group), corresponding to the gross and imaging findings. E) Female KO mice with a phenotype of infertility demonstrate that the tissue of the grossly smaller KO ovary contains markedly fewer (or no) follicles than the ovary of WT mice on representative H&E sections. F) The number of follicles is significantly smaller in the KO ovary than in the WT ovary (n = 10 mice per group). Scale bars = 100 μm. Values are means ± SE. *P < 0.05, **P < 0.001, ***P < 0.0001.

Under basal conditions, morphological examinations between the two groups of 12-week-old mice showed that *WNT10A*^–/–^mice were grossly smaller in size than WT mice and had overt alopecia and kyphosis (Fig 1A). Whole-body X-ray confirmed growth retardation in *WNT10A*^–/–^mice, accompanied by severe kyphosis and many hyperlucent areas in the bone (Fig 1B). Indeed, at 1 to 12 months of age, both male and female *WNT10A*^–/–^mice had significantly lower body weights than WT mice (Fig 1C). Quantitative analyses demonstrated that the bone length and BMD in both male and female 12-week-old *WNT10A*^–/–^mice were significantly lower than those of WT mice (bone length: *P <* 0.05; BMD: *P <* 0.0001) (Fig 1D). Furthermore, female homozygous *WNT10A*^–/–^mice showed a phenotype of infertility, since the tissue of the *WNT10A*^–/–^ovaries histopathologically had no or markedly fewer follicles than the ovaries in WT mice (Fig 1E and 1F).

Collectively, our *WNT10A*^–/–^mice showed a range of unique phenotypes of morpho/organogenetic failure, including growth retardation, alopecia, kyphosis and infertility, in a background of essentially stromagenic failure, including osteogenetic and follicle growth failure.

### Deletion of WNT10A resulted in the delayed wound healing associated with suppressed stromagenesis, especially in fibroblasts/myofibroblasts

**Fig 2:**
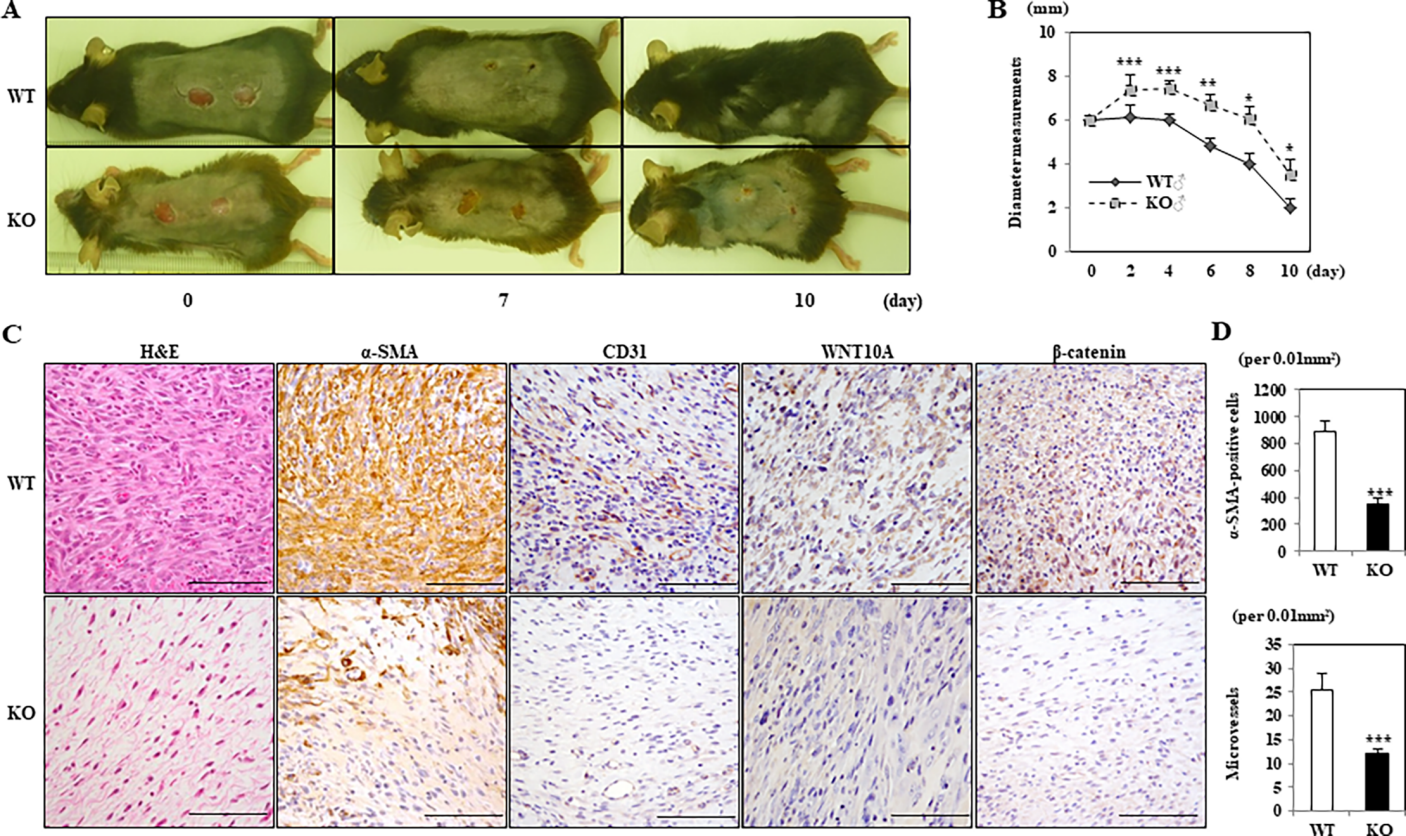
*WNT10A*^–/–^KO mice showing the delayed skin ulcer/wound healing associated with suppressed stromagenesis, especially in fibroblasts/myofibroblasts. **A)** Grossly, KO mice display significantly larger injured areas of skin along with more delayed wound healing than WT mice (n = 8 mice per group) within 10 days of observation of this murine *in vivo* model. Interestingly, WT mice at day 10 post-injury showed new hair growth, whereas KO mice did not. **B)** Accordingly, the diameters of the KO skin wound were significantly longer than those of WT mice (n = 8 mice per group) from days 2 to 10 post-injury. **C)** The representative histopathological pictures reveal that the inflammatory/edematous granulation tissue in KO skin ulcer (bottom) is significantly larger and less cellular (H&E), accompanied by more decreased number of α-SMA-positive spindle fibroblasts/myofibroblasts, than that in WT mice (upper) (n = 8 mice per group), only in which not only WNT10A but β-catenin expression is immunohistochemically overt especially in those activated (myo)fibroblasts. In addition, the incurable KO skin wound contains significantly more repressed and fewer CD31-positive microvessels than that of WT mice. Scale bars = 100 μm. **D)** Correspondingly, there are significantly fewer α-SMA-positive fibroblasts/myofibroblasts and CD31-positive microvessels in KO mice than in WT mice (n = 8 mice per group). Values are means ± SE. *P < 0.05, **P < 0.001, ***P < 0.0001.

**Fig 3:**
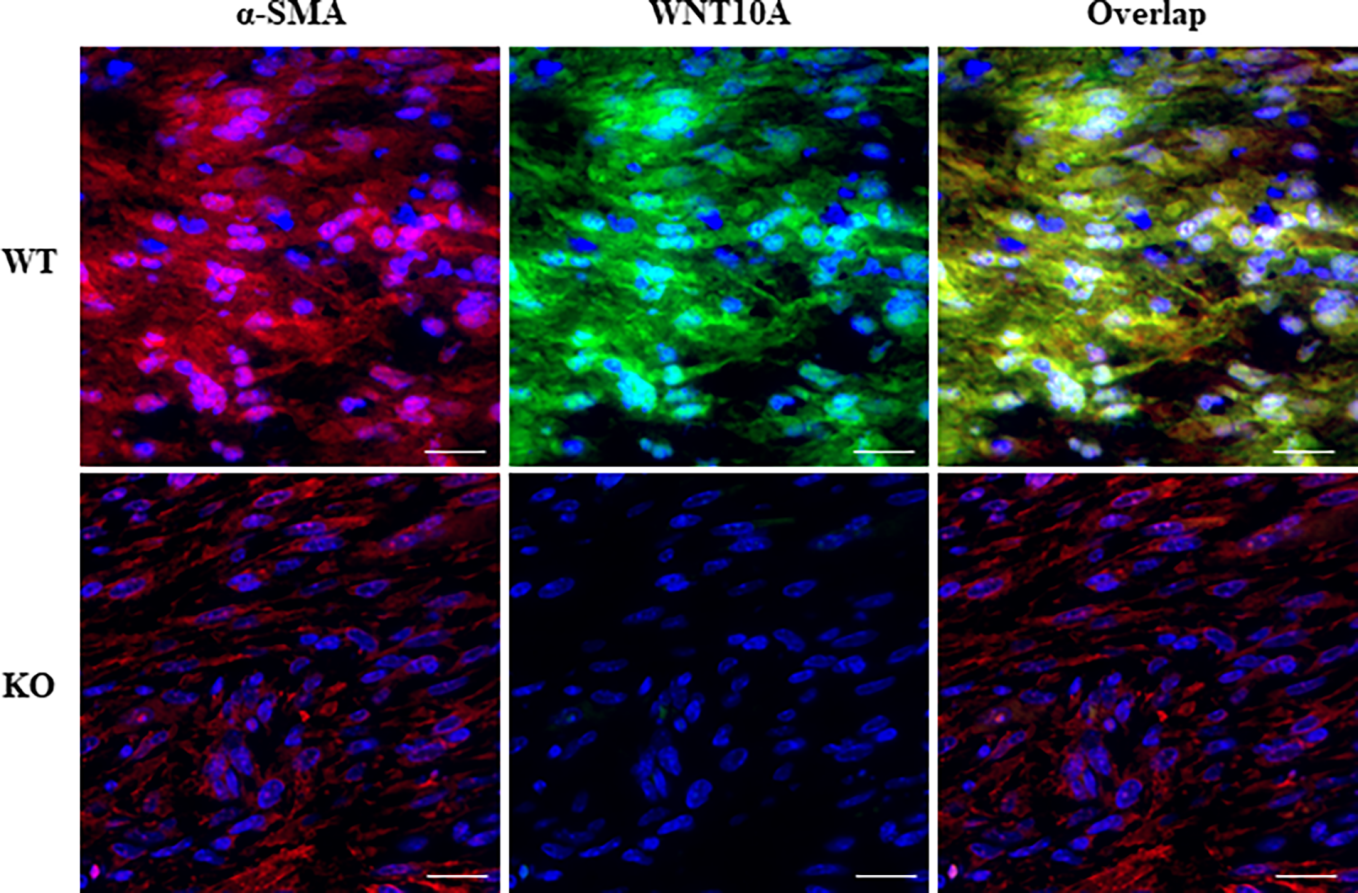
Specific WNT10A expression in the *WNT10A*^+/+^ WT mice activated fibroblasts/myofibroblasts in the murine wound healing model. Double-immunofluorescence staining show that a large number of α-SMA-positive fibroblasts/myofibroblasts (*red-stained*) in the WT skin wounds (upper) had specific expression of WNT10A (*green-stained*), whereas those of KO mice did not (bottom) (n = 8 mice per group). Scale bars = 20 μm.

We subjected WT and *WNT10A*^–/–^mice to skin ulcer formation in a murine model of wound healing. Regarding the gross appearance within 10 days, *WNT10A*^–/–^mice showed significantly larger injured areas along with more delayed wound healing compared to WT mice (Fig 2A). Interestingly, WT mice at day 10 post-injury demonstrated new hair growth, but *WNT10A*^–/–^mice not (Fig 2A). A quantitative analysis revealed that the diameter of the *WNT10A*^–/–^skin wound was significantly longer than that in WT mice from days 2 to 10 post-injury (day 2 to 4: *P <* 0.0001; day 6: *P <* 0.001; day 8 to 10: *P <* 0.05) (Fig 2B). A microscopic examination showed that the inflammatory/edematous granulation tissue in *WNT10A*^–/–^skin ulcer was significantly larger and less cellular and accompanied by less expression ofα-SMA and more decreased number of α-SMA-positive spindle fibroblasts/myofibroblasts (Fig 2C) than that in WT mice, only in which not only WNT10A but β-catenin expression were overt especially in those activated (myo)fibroblasts (Fig 2C). In addition, the incurable *WNT10A*^–/–^skin wound contained significantly more repressed and fewer CD31-positive microvessels (Fig 2C). Indeed, the numbers of α-SMA-positive fibroblasts/myofibroblasts and CD31-positive microvessels were significantly lower in *WNT10A*^–/–^mice than in WT mice (fibroblasts/myofibroblasts: WT 883.7 ± 89.0 per 0.01 mm^2^ vs. *WNT10A*^–/–^ 354.6 ± 37.0 per 0.01 mm^2^; *P <* 0.0001; and microvessels: WT 25.4 ± 3.7 per 0.01 mm^2^ vs. *WNT10A*^–/–^ 12.0 ± 1.1 per 0.01 mm^2^; *P <* 0.0001) (Fig 2D). Double-immunofluorescence staining (Fig 3) ultimately confirmed that these WNT10A-expresseing spindled cells (*green-stained*) were α-SMA-positive fibroblasts/myofibroblasts (*red-stained*) in the skin wounds of WT mice, but completely not in the skin wounds of *WNT10A*^–/–^mice.

**Fig 4:**
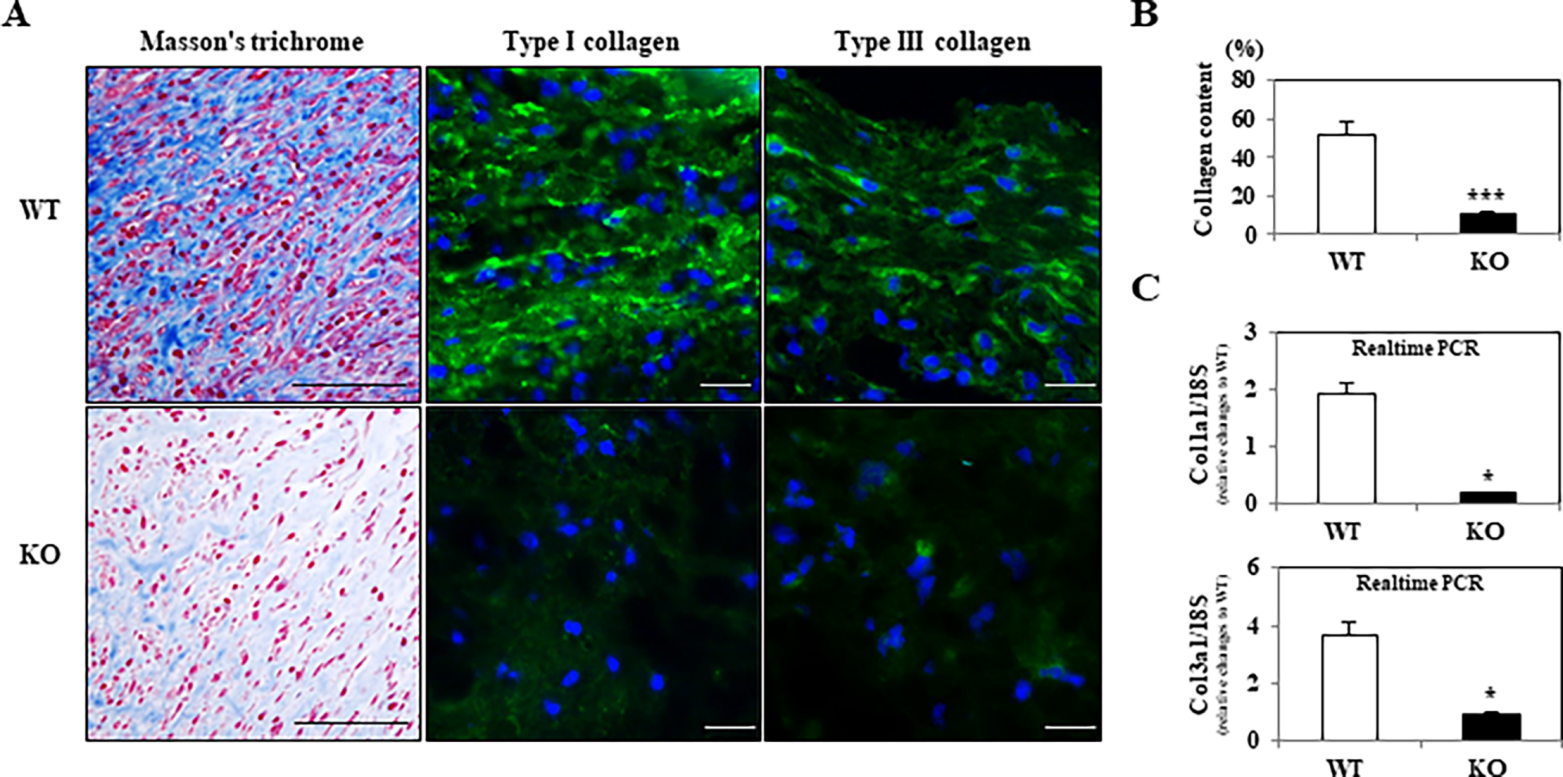
*WNT10A*^–/–^KO skin wound showing reduced fibrosis/fibrogenesis associated with the decreased expression of stromagenesis-related genes. **A)** Representative pictures of Masson’s trichrome staining (Scale bars = 100 μm) show that collagen deposits (*blue*-stained) were markedly smaller with more reduced fibrosis in the injured incurable skin lesions of KO mice than in those of WT mice (n = 8 mice per group). In addition, an immunofluorescence study (Scale bars = 20 μm) reveals that a large number of WT spindle (myo)fibroblasts (*blue-stained* in nuclei) have significant expression of Type I/III collagen at day 10 post-skin injury, which is very rarely or not observed in KO (myo)fibroblasts. **B)** On a quantitative analysis, the *blue*-stained collagen content of KO injured skin was found to be markedly lower than in WT skin. **C)** Correspondingly, real-time RT-PCR showed that the mRNA expression of *Type I* and *Type III collagen* was significantly lower in the KO skin of the day-3 wound healing model than in the WT skin. Values are means ± SE and were normalized for 18s rRNA expression (real-time RT-PCR). *P < 0.05, ***P < 0.0001.

Furthermore, in order to calculate the ratio of collagen content to granulation, as shown in Fig 4A, we performed Masson’s trichrome staining, revealing that the collagen deposits (*blue*-stained) in areas of reduced fibrosis in injured skin lesions were smaller in *WNT10A*^–/–^mice than in WT mice (WT 51.9% ± 6.1% vs. *WNT10A*^–/–^ 10.1% ± 1.5%; *P <* 0.0001) (Fig 4B). An immunofluorescence study demonstrated that a large number of spindle (myo)fibroblasts (*blue-stained* in nuclei) had significant expression of Type I/III collagen (*green-stained*), especially in WT mice at day 10 post-skin injury; in contrast, this expression was very rarely observed in *WNT10A*^–/–^mice (Fig 4A). Corresponding to those data, real-time RT-PCR showed that the mRNA expression of *Type I* and *Type III collagen* was significantly lower in the skin of day-3 wound healing model *WNT10A*^–/–^mice than in that of WT (*P* < 0.05 and *P <* 0.05, respectively) (Fig 4C) and control mice (*P* < 0.05 and *P <* 0.01, respectively) (data not shown).

cDNA expression array analyses were performed comparing non-treated control as well as day-3 post-injury skin tissue samples obtained from *WNT10A*^–/–^and *WNT10A*^+/+^ WT mice. A total of 19 genes showed 2-fold down-regulation in control *WNT10A*^–/–^mice. Those genes are shown in Table 1. These findings included the apparent down-regulation of *Collagen Type I*, *Type III* and *Type V*. Three genes still showed down-regulation in the day-3 post-injury skin tissue samples obtained from *WNT10A*^–/–^mice and *WNT10A*^+/+^ WT mice: *Collagen Type I*, *Type III* and *WNT10A*.

**Table 1:** Expression of extracellular matrix associated genes in skin tissue by microarray studies.

Gene Symbol	Accession number	KO/WT cont.	KO/WT 3D	Gene description
Ahsg	NM_013465	**0.09**	1.43	alpha-2-HS-glycoprotein
Ccdc80	NM_026439	**0.38**	0.74	coiled-coil domain containing 80
Col1a1	NM_007742	**0.48**	**0.53**	collagen, type I, alpha 1
Col3a1	NM_009930	**0.67**	**0.21**	collagen, type III, alpha 1
Col5a1	NM_015734	**0.46**	1.1	collagen, type V, alpha 1
Col5a3	NM_016919	**0.28**	1.03	collagen, type V, alpha 3
Col15a1	NM_009928	**0.45**	0.83	collagen, type XV
Dpt	NM_019759	**0.47**	1.09	dermatopontin
Fn1	NM_010233	**0.49**	2.03	fibronectin 1
Lgals3bp	NM_011150	**0.45**	0.94	lectin, galactoside-binding, soluble, 3 binding protein
Nid2	NM_008695	**0.29**	1.21	nidogen 2
Ogn	NM_008760	**0.33**	0.58	osteoglycin
Nid1	NM_010917	**0.48**	1.2	nidogen 1
Adamts5	NM_011782	**0.39**	1.38	a disintegrin-like and metallopeptidase (reprolysin type) with thrombospondin type 1 motif, 5 (aggrecanase-2)
Spon2	NM_133903	**0.4**	0.73	spondin 2, extracellular matrix protein
Tgm2	NM_009373	**0.45**	1.04	transglutaminase 2, C polypeptide
Wnt10a	NM_009518	**0.06**	**0.05**	wingless related MMTV integration site 10a

### *WNT10A*-deficiency suppressed collagen expression and production in fibroblasts/myofibroblasts

**Fig 5:**
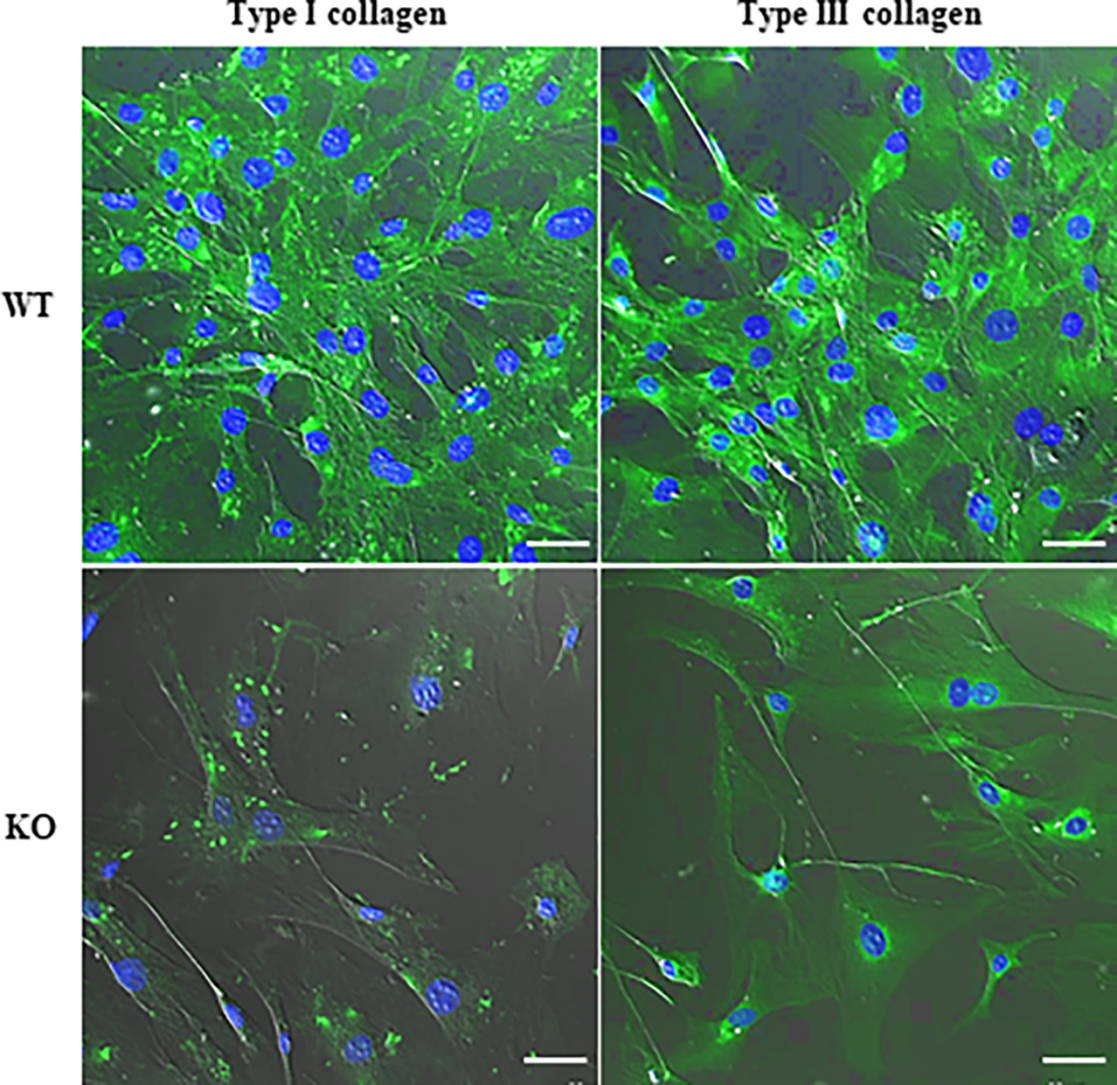
Immunofluorescence study of *in vitro* murine dermal fibroblasts showing a unique phenotype of little stromagenesis on *WNT10A*^–/–^KO mice. Immunofluorescence staining shows that a significantly larger number of cultured dermal fibroblasts (*blue-stained* in nuclei) contain elevated expression of Type I/III collagen (*green-stained*) in WT mice than in KO mice (n = 3 per group). The basal condition of fibroblastic proliferation is overtly better in WT mice than that in KO mice, and the morphology of fibroblasts from KO skin is relatively poorly preserved. Scale bars = 50 μm.

**Fig 6:**
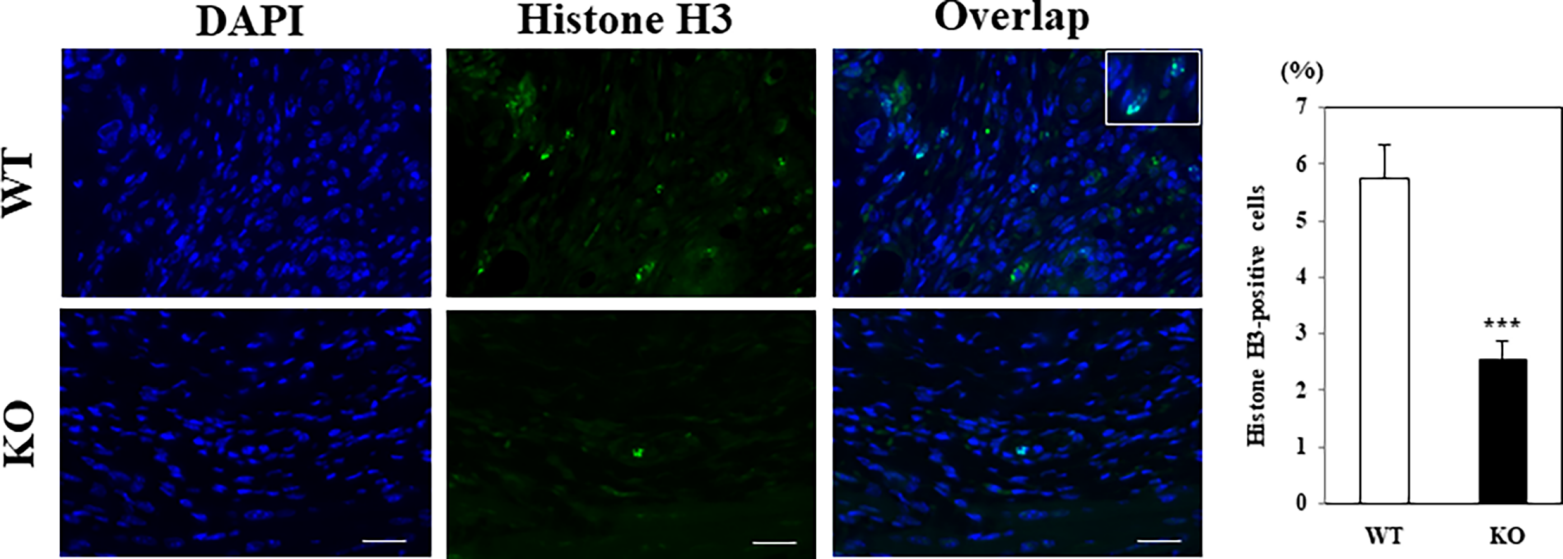
The deficiency of *WNT10A* suppressed cell proliferation activity in wound healing mice model. Immunofluorescence staining showed that a significantly larger number of histone H3-positive cells (*green-stained*) were observed in WT mice wound skin, compared to *WNT10A*^–/–^mice (n = 3 per group). The fluorescence of histone H3 was located in nuclei (inset) (*blue-stained* in nuclei). Scale bars = 100 μm.

Immunofluorescence staining showed that a significantly fewer number of cultured *WNT10A*^–/–^dermal fibroblasts (*blue-stained* in nuclei) expressed Type I/III collagen (*green-stained*) than in dermal fibroblasts from WT mice (Fig 5). Since WNT10A is a secreted protein it should be in conditioned medium (CM) from WT cells but not in CM from WNT10A-/- cells. Therefore CM from WT cells should be able to increase expression of Col1a1 and Col3a1 in WNT10A-/- cells. To test this we produced CM from WT cells and applied it to WNT10A-/- cells. As expected, Type I/III collagen transcript expression can be increased by conditioned medium in cultured dermal fibroblasts isolated from *WNT10A*^–/–^mice (S4 Fig). The basal condition of fibroblastic proliferation was overtly worse in *WNT10A*^–/–^mice than in WT mice, and the morphology of fibroblasts from *WNT10A*^–/–^skin was relatively poorly preserved (Fig 5). Moreover, histone H3 staining also showed that proliferation activity of *WNT10A*^–/–^dermal fibroblasts/myofibroblasts was significantly weaker than fibroblasts/myofibroblasts from WT mice (Fig 6), even though not carried out over several time points.

## Discussion

The current study indicates, for the first time, the critical and central *in vivo* functions of WNT10A in ‘wound’ healing with collagen expression/synthesis, particularly in our *WNT10A*^–/–^mice. First, under basal condition, *WNT10A*^–/–^mice uniquely showed various phenotypes of morpho/organogenetic failure, such as growth retardation, alopecia, kyphosis and infertility, in a background of osteogenetic and follicle growth failure. These features are likely in agreement with human phenotypes of *WNT10A* mutations, such as odonto-onycho-dermal dysplasia, a very rare autosomal recessive ectodermal dysplasia syndrome characterized by sparse hair, severe tooth agenesis and onychodysplasia [33]. Furthermore, another group has reported that *WNT10A*-null mice showed severe tooth agenesis with molar crown and root dysmorphogenesis [34]. More recently, Xu *et al*. have also confirmed that *WNT10A*^–/–^mice display a phenotype of tooth defects, but, in contrast to us, demonstrate no apparent failure of embryonic development on skin appendages including hair follicles [35]. Taken together, these findings in *WNT10A*^–/–^mice suggest that the WNT10A signal transduction system might play a central, key role in many processes of morpho/organogenesis, although further in-depth molecular analyses are needed to confirm these findings and their potential association with stem cell biology.

**Fig 7:**
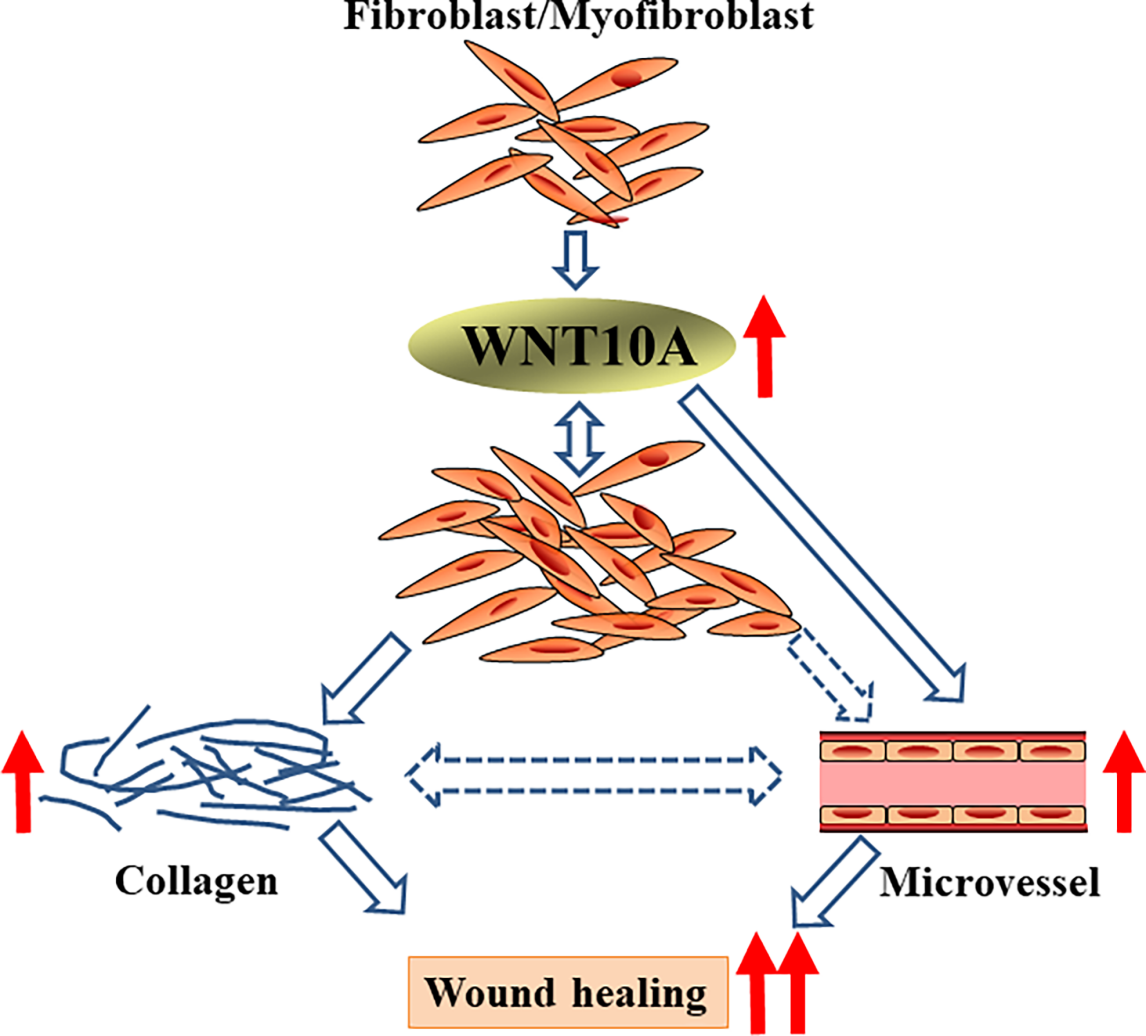
A schematic presentation of the critical *in vivo* roles of WNT10A in ‘wound’ healing. This diagram depicts the central, key roles of WNT10A in the current murine model of wound healing. Our obtained data confirm that WNT10A expression, especially in fibroblasts/myofibroblasts, can be crucially, responsible for various potentially beneficial effects against wound healing.

Second, skin injury-induced fibrosis/fibrogenesis and angiogenesis with collagen expression, was significantly reduced and wound healing was consequently delayed in *WNT10A*^–/–^mice compared with WT mice. *WNT10A*^–/–^mice showed potential anti-healing profiles associated with fibrogenic failure and less ECM (collagen) expression/production, especially in the acute to subacute phase within the first 10 days post-injury. Our present data suggest that WNT10A-deficiency plays a pivotal role in wound healing, along with (i) the decreased number of (myo)fibroblasts, at least in part, via reduced neovascularization; and (ii) the decreased synthesis of collagen-rich ECM via repressed collagen and β-catenin expression. A diagram depicting the central, key functions and roles of WNT10A in wound healing is shown in Fig 7. All of these features imply that specific activators of the WNT10A signaling pathway and WNT10A agonists might offer therapeutic strategies against various human injuries, including postoperative skin wound. However, the clinical relevance of these drugs must be verified in the future in order to prevent expected or unexpected adverse reactions. Overall, our obtained data have confirmed that WNT10A expression, especially in the fibroblasts and/or myofibroblasts, is crucially responsible for various potentially beneficial effects against wound healing, as shown in Fig 7.

In line with our previous data [15], after establishing the murine wound healing model, we detected the specific expression of WNT10A in the injured skin ulcer of WT mice by immunohistochemistry, but not in *WNT10A*^–/–^mice. It has been reported that the canonical WNT10A/β-catenin signaling pathway is most likely stimulated by distinct mechanisms during the tissue repair process and fibrosis through potential fibrogenesis with collagen expression in response to various immune system mediators, such as tumor necrosis factor (TNF)-α or nuclear factor (NF)-κB [3, 13, 36], and/or various oxidative stressors [15, 17]. The activation of WNT10A signaling subsequently stabilizes and increases the expression of downstream β-catenin [8, 12]. Indeed, the expression of β-catenin was significantly lower in *WNT10A*^–/–^injured skin than in that of WT mice in the post-wound healing model.

These present data are in agreement with various previous observations from our laboratories: (a) In *in vivo* nude mouse xenograft models with injection of HeLa cells, WNT10A overexpression significantly promotes the proliferation of both microvascular endothelial cells and fibroblasts and/or myofibroblasts [15]; (b) the specifically positive expression of WNT10A is recognized, especially in the activated, α-SMA-positive dermal fibroblasts/myofibroblasts within the histopathology of human keloid, but not in that of normal skin dermal fibrocytes [15]; (c) WNT10A-expressing kidney fibroblasts significantly induce kidney fibrosis and correlate with kidney dysfunction in human acute interstitial nephritis [17]; and (d) WNT10A overexpression has a close relationship with the acute exacerbation and poor prognosis of idiopathic pulmonary fibrosis [18]. In this context, the WNT10A signaling pathway might be similar to that of apoptosis signal-regulating kinase 1 (ASK1), a mitogen-activated protein kinase that is also ubiquitously-expressed and situated in an important upstream position for many signal transduction pathways [21, 26, 27]. Indeed, our groups have shown that *ASK1*-deficient mice display anti-healing phenotypes as well as (i) the reduced migration of smooth muscle cells (SMCs) into neointima via decreased neovascularization and (ii) the decreased synthesis of collagen-rich ECM, at least in part, via suppressed dedifferentiation into synthetic SMCs [26]. In addition, the ASK1-dependent signaling pathway is considered to be significantly correlated with fibrogenesis/angiogenesis and collagen expression, in various human diseases, from injury to neoplasia [37, 38]. Further follow-up with in-depth experiments is therefore needed to investigate the relationship between WNT10A and ASK1. But mechanism(s) for how WNT10A regulates collagen expression can also be left for another research article.

In conclusion, based on our collected data of *WNT10A*-deficient mice, we postulate that a) loss of WNT10A is not lethal but that mice are smaller, have kyphosis, and are female sterile; b) wound healing does not occur normally in *WNT10A*^–/–^mice; c) collagen and b-catenin expression are disrupted wounds from *WNT10A*^–/–^mice. Ultimately, WNT10A signaling can play a critical in vivo role in wound healing by regulating the expression and synthesis of collagen.

## Supporting information

S1 Fig Schema of human WNT10A gene construct and the PCR primer sit. (PPTX)

S2 Fig The genes expression patterns in WNT10A gene knockout mice. RT-RCR showed that no WNT10A expression but LacZ and Neo expressions was observed in homozygous mice, whereas only WNT10A expression was found in WT mice, while all three genes expressions were confirmed in heterozygous mice.
(PPTX)

S3 Fig The *WNT10A* expression in mice skin tissues. RT-RCR (A) and western blotting (B) showed the deficiency of WNT10A mRNA and protein expression were confirmed in *WNT10A*^–/–^mice skin (n = 3 mice per group).
(PPTX)

S4 Fig The *in vitro* role of WNT10A in collagen expression in murine dermal fibroblasts. Conditioned medium (the culture medium of dermal fibroblasts isolated from WT mice skin) promoted type I and III collagen expression in fibroblasts isolated from *WNT10A*^–/–^mice skin (n = 3 mice per group). Values are means ± SE. *P < 0.05, ***P < 0.0001.
(PPTX)

## Abbreviations

*WNT10A*^–/–^: *WNT10A*-deficient mice
ECM: extracellular matrix
α-SMA: α-smooth muscle actin
BMD: bone mineral density
