# Supplementary figures and images for: Critical in vivo roles of WNT10A in wound healing by regulating collagen expression/synthesis in WNT10A-deficient mice
Source: PLoS One. 2018 Mar 29;13(3):e0195156. doi: 10.1371/journal.pone.0195156 (PMC5875851; doi:10.1371/journal.pone.0195156)

## Slide 1
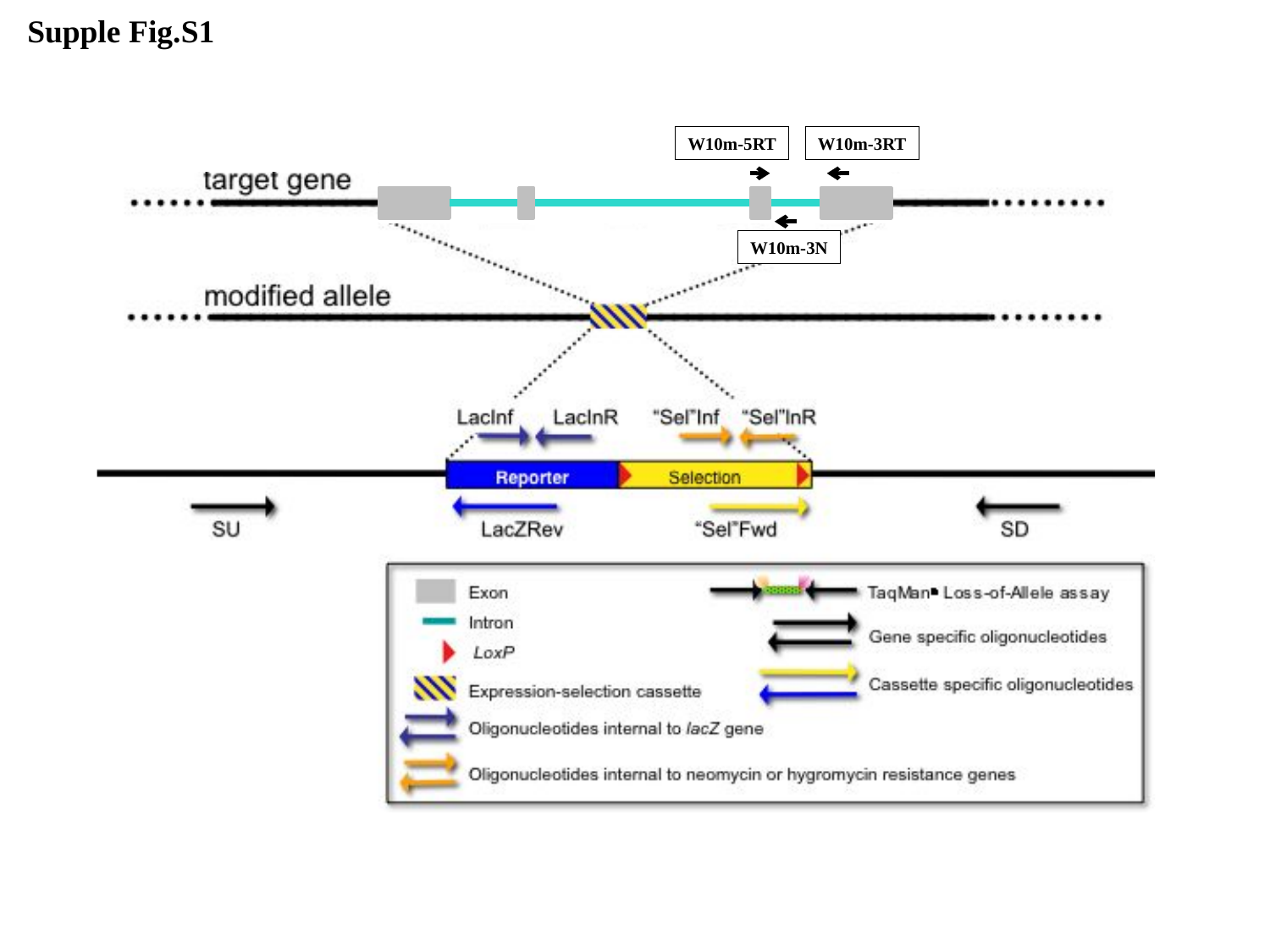

Supple Fig.S1
W10m-5RT
W10m-3RT
W10m-3N

Supplement: S1 Fig — (PPTX) [file pone.0195156.s001.pptx]

## Slide 1
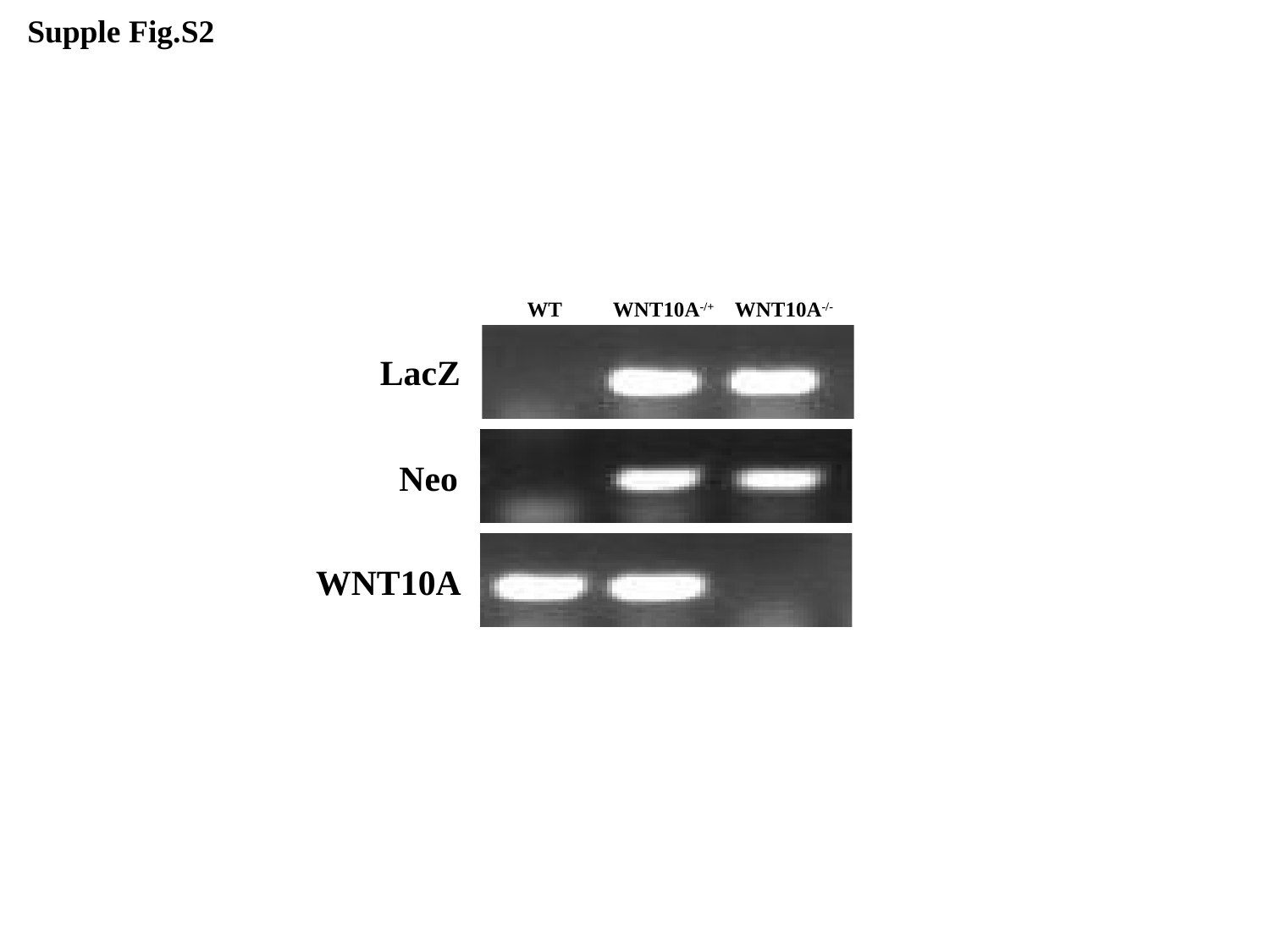

Supple Fig.S2
WT
WNT10A-/+
WNT10A-/-
LacZ
Neo
WNT10A

Supplement: S2 Fig — RT-RCR showed that no WNT10A expression but LacZ and Neo expressions was observed in homozygous mice, whereas only WNT10A expression was found in WT mice, while all three genes expressions were confirmed in heterozygous mice. (PPTX) [file pone.0195156.s002.pptx]

## Slide 1
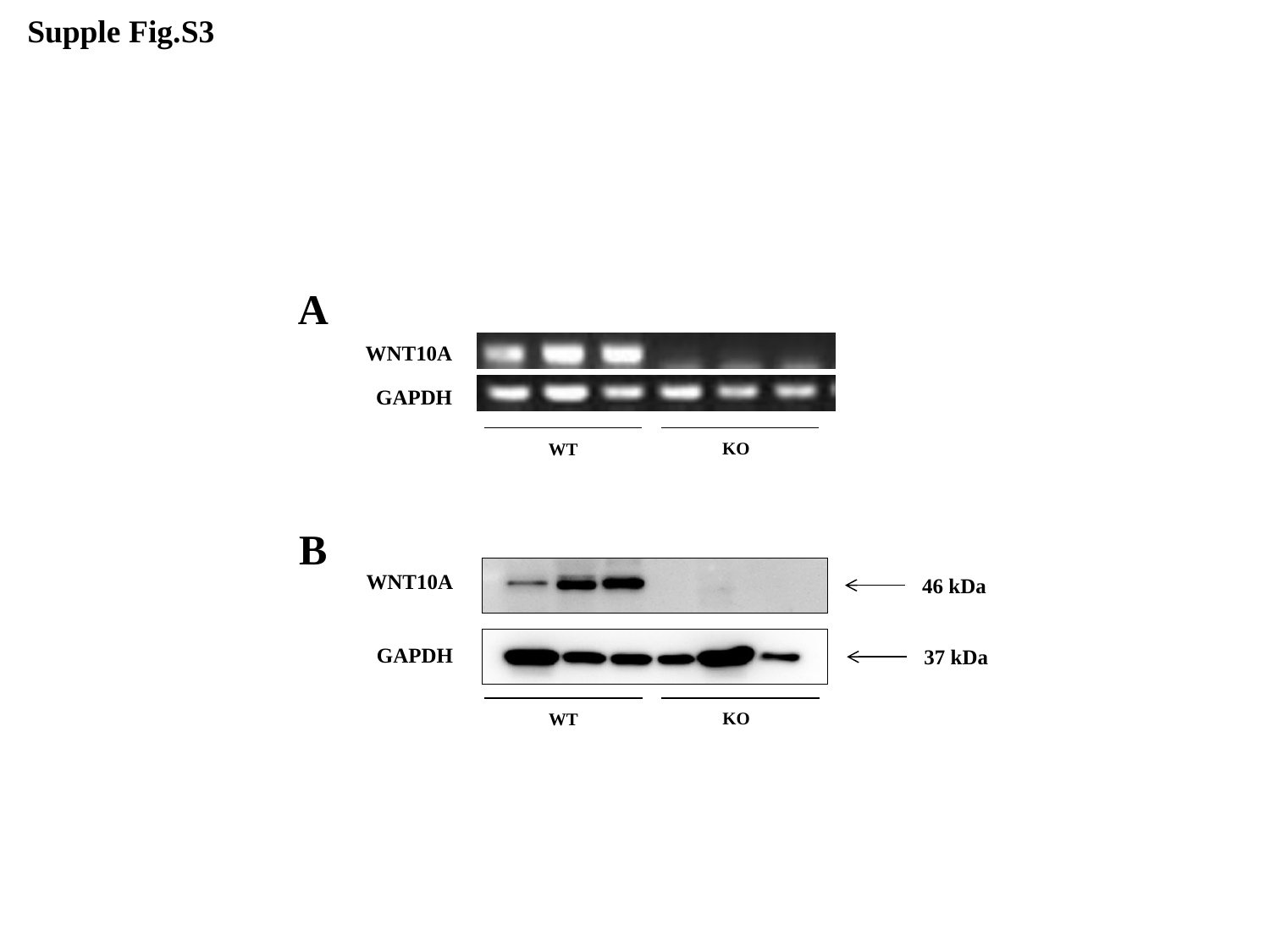

Supple Fig.S3
A
WNT10A
GAPDH
KO
WT
B
WNT10A
46 kDa
GAPDH
37 kDa
KO
WT

Supplement: S3 Fig — RT-RCR (A) and western blotting (B) showed the deficiency of WNT10A mRNA and protein expression were confirmed in WNT10A–/–mice skin (n = 3 mice per group). (PPTX) [file pone.0195156.s003.pptx]
