# Supplementary material for: Critical in vivo roles of WNT10A in wound healing by regulating collagen expression/synthesis in WNT10A-deficient mice
Source: PLoS One. 2018 Mar 29;13(3):e0195156. doi: 10.1371/journal.pone.0195156 (PMC5875851; doi:10.1371/journal.pone.0195156)
Supplement: S4 Fig — Conditioned medium (the culture medium of dermal fibroblasts isolated from WT mice skin) promoted type I and III collagen expression in fibroblasts isolated from WNT10A–/–mice skin (n = 3 mice per group). Values are means ± SE. *P < 0.05, ***P < 0.0001. (PPTX) [file pone.0195156.s004.pptx]

## Slide 1
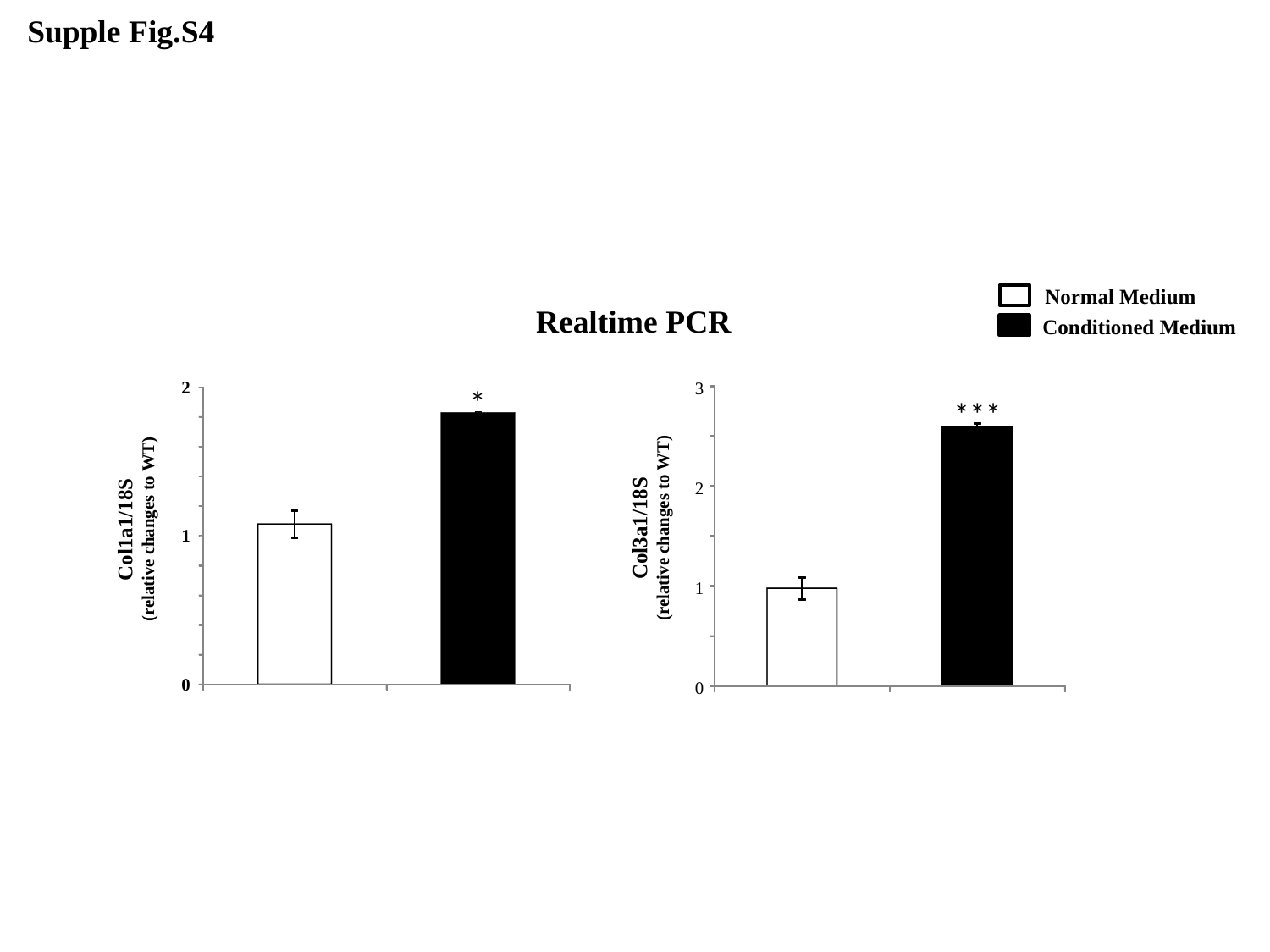

Supple Fig.S4
Normal Medium
Realtime PCR
Conditioned Medium
*
2
1
0
3
2
1
0
***
Col3a1/18S
(relative changes to WT)
Col1a1/18S
(relative changes to WT)
